# Crystal Structure of *Bacillus cereus* HlyIIR, a Transcriptional Regulator of the Gene for Pore-forming Toxin Hemolysin II

**DOI:** 10.1016/j.jmb.2006.10.074

**Published:** 2007-01-19

**Authors:** Oleg V. Kovalevskiy, Andrey A. Lebedev, Alexei K. Surin, Alexander S. Solonin, Alfred A. Antson

**Affiliations:** 1 Institute of Biochemistry and Physiology of Microorganisms, Russian Academy of Sciences, Pushchino, Moscow Region, 142290, Russia; 2 Structural Biology Laboratory, Department of Chemistry, University of York, Heslington, York, YO10 5DD, UK; 3 Institute of Protein Research, Russian Academy of Sciences, Pushchino, Moscow Region, 142290, Russia

**Keywords:** HlyII, hemolysin II, RNAP, RNA-polymerase, SELEX, systematic evolution of ligands by exponential enrichment, SeMet, selenomethionine, HlyIIR, X-ray crystallography, DNA-binding, TetR family, hemolysin II

## Abstract

Production of *Bacillus cereus* and *Bacillus anthracis* toxins is controlled by a number of transcriptional regulators. Here we report the crystal structure of *B. cereus* HlyIIR, a regulator of the gene encoding the pore-forming toxin hemolysin II. We show that HlyIIR forms a tight dimer with a fold and overall architecture similar to the TetR family of repressors. A remarkable feature of the structure is a large internal cavity with a volume of 550 Å^3^ suggesting that the activity of HlyIIR is modulated by binding of a ligand, which triggers the toxin production. Virtual ligand library screening shows that this pocket can accommodate compounds with molecular masses of up to 400–500 Da. Based on structural data and previous biochemical evidence, we propose a model for HlyIIR interaction with the DNA.

## Introduction

Bacteria of the *Bacillus cereus* group, including *B. cereus, B. anthracis, B. weihenstephanensis* and *B. thuringiensis* are recognized as important human and animal pathogens. Their pathogenic properties are distinct, e.g. *B. anthracis* is a strong mammalian pathogen and the etiologic agent of anthrax, *B. thuringiensis* is pathogenic to insects while *B. cereus* is an opportunistic human pathogen. Nevertheless, genomic and rRNA analysis of members of this taxa reveals only small sequence variations that would normally be expected for different strains of a single bacterial species.^1^ The significant differences in the pathogenic properties of bacteria of the *B. cereus* group are due to the production of different sets of toxins, some of which are encoded by plasmid-borne genes.

*B. cereus* is a Gram-positive, spore-forming rod-like bacterium, commonly found in soil, water and as a contaminant in food and pharmaceutical products.^2,3^ This microorganism causes emetic and enteric food-poisoning, periodontitis and systemic infections occurring as a consequence of traumatic injuries.^4^ The pathogenic properties of *B. cereus* are determined by the production of several extracellular virulence factors. Synthesis of these protein toxins is subject to genetic regulation. To date, the following regulators involved in the control of expression of virulence factors in *B. cereus* were characterized: (1) PlcR, a global transcriptional regulator controlling expression of tens of genes^5,6^; (2) Fur, a ferric uptake repressor responsible for iron metabolism regulation and required for full virulence;^7^ and (3) HlyIIR, a specific regulator of the hemolysin II (*hlyII*) gene.^8^ PlcR was initially described as a positive regulator of the phospholipase C gene, but later studies showed that it directly controls more than 100 genes and operons.^5,6^ In particular, production of most protein toxins of *B. cereus* is positively regulated by PlcR. Another global regulator, Fur, controls genes responsible for iron uptake and storage by binding to its DNA recognition site (Fur-box) in a metal-dependent manner.^7^ Recent studies using an insect infection model demonstrated that a *fur* null-mutant of *B. cereus* is significantly attenuated.^7^ A Fur-box was found in the promoter regions of cytotoxin K and *hlyII* genes. In the case of the *hlyII* gene, the Fur binding site overlaps with the transcription start point suggesting that Fur binding must repress *hlyII* expression. It is interesting to note that unlike most other cytotoxins of *B. cereus*, *hlyII* is not regulated by PlcR; recent studies showed that transcription of the *hlyII* gene is instead regulated by HlyIIR.^8^

HlyII belongs to the family of oligomeric β-barrel channel-forming toxins. It shares significant sequence homology (31% identity) with the alpha-toxin, a major pathogenicity factor of *Staphylococcus aureus*, for which the three-dimensional structure is available.^9^ HlyII can lyse various kinds of eukaryotic cells although its cytolytic activity against mammalian erythrocytes depends on the particular species.^10^ It is still not clear why *hlyII* gene expression is so tightly controlled, with at least one global regulator (Fur) and also one specific regulator (HlyIIR).

The hemolysin II regulator gene, *hlyIIR*, is located immediately downstream of *hlyII* and its product regulates *hlyII* expression by specifically binding to a 44 bp perfect inverted DNA repeat (22 bp × 2), centred 48 bp upstream of the *hlyII* transcription initiation point. HlyIIR negatively regulates expression from the *hlyII* promoter in heterologous system in *Escherichia coli* cells.^8^ It was also shown to inhibit *hlyII* transcription *in vitro*, using both *E. coli* and *B. cereus* RNA-polymerase (RNAP), by interfering with the process of isomerisation of the RNAP closed promoter complex into the catalytically active open promoter complex. In addition, HlyIIR is able to interact with RNAP (both core and holoenzyme) in solution.^8^ Recent biochemical studies indicated that HlyIIR exists as a dimer in solution and two such dimers bind to *hlyII* operator DNA; circular dichroism spectra suggested no significant conformational changes in the operator DNA upon binding to HlyIIR (Rodikova *et al*., unpublished results).

The polypeptide chain of HlyIIR contains 201 amino acid residues and has a molecular mass of 23.5 kDa. Amino acid sequence analysis^11^ indicated that HlyIIR exhibits distant homology to the TetR family of transcription regulators, with the N-terminal amino acid segment 12–58 of HlyIIR aligning with the N-terminal helix-turn-helix domain of TetR. The closest HlyIIR homologues are uncharacterised putative TetR family regulators from *Polaromonas sp.* JS666 (31% sequence identity for a 144 residue segment of YP_550862), *Marine alpha proteobacterium* (27% sequence identity for a 201 residue segment of AAR21626) and *Rhizobium etli* (31% sequence identity for a 159 residue segment of YP_472143). In general, the N-terminal domain of HlyIIR exhibits high sequence homology to members of the TetR family while its C-terminal portion appears to be divergent.

Although HlyIIR has been characterised biochemically, to date little is known about its exact three-dimensional organisation except for limited information that could be inferred from distant homologues of known structure (∼ 20% sequence identity). It was also unclear how its repressor activity is modulated. Here we address these issues by reporting the crystal structure of *B. cereus* HlyIIR. The structure reveals an unexpectedly large internal cavity that could accommodate a ligand with the molecular mass of up to 500 Da, suggesting a mechanism for triggering the expression of hemolysin II.

## Results

### Overall structure of HlyIIR

**Figure 1:**
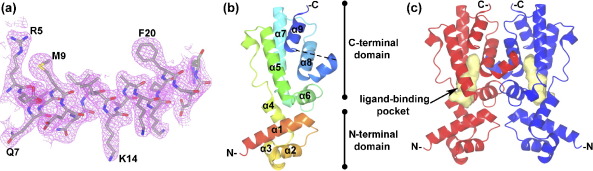
The HlyIIR structure. (a) Electron density corresponding to one of the protein helices (α1) calculated with maximum likelihood weighted coefficients 2|*F*_o_|–|*F*_c_| and contoured at 1.25σ. (b) and (c) Ribbon diagrams of HlyIIR. Monomer (b) is rainbow-coloured with its N-terminal in red and C-terminal in blue. Dotted line indicates the disordered segment, which was not modelled. The biological dimer (c) is generated by the crystallographic 2-fold axis. The large internal cavity (yellow) is drawn along the van der Waals radii of cavity-forming residues. The cavity surface was calculated by SURFNET.^33^ This Figure and Figures 3 and 4 were prepared using CCP4MG.^34^

The structure was determined by multi-wavelength anomalous dispersion (MAD) with selenomethionine (SeMet) HlyIIR (Table 1; Figure 1), since a molecular replacement approach with the closest structural homologue (*E. coli* Ycdc protein; PDB code 1PB6; 22% overall sequence identity) did not lead to structure solution. In the final model refined at 2.4 Å resolution all residues are within the most favoured and additionally allowed regions of the Ramachandran plot, with all stereochemical values within or better than the expected range.^12^ The model contains 179 amino acid residues (Ser4–Lys198) except for a segment of 16 residues (Leu170–Glu185) for which there was no clear electron density.

The asymmetric unit contains a single monomer of HlyIIR, whilst the protein exists as a homodimer *in vitro* and *in vivo* (Rodikova *et al*., unpublished results). In the crystal structure, the biological dimer is generated by the crystallographic 2-fold axis. The dimer of HlyIIR has an Ω shape, typical for the TetR family of repressors, with overall dimensions of about 60 Å × 60 Å × 25 Å.

HlyIIR has an all α-fold, with each subunit consisting of nine α-helices. The tertiary structure is stabilised by hydrophobic contacts between helices and by 11 salt bridges. A monomer of HlyIIR can be subdivided into a small N-terminal DNA-binding domain and a larger C-terminal dimerisation domain. (Figure 1(b) and (c)). These two domains are associated with each other through a shared hydrophobic core and two salt bridges (Glu26–Arg114 and Lys17–Glu99).

### N-terminal DNA-binding domain

**Figure 2:**
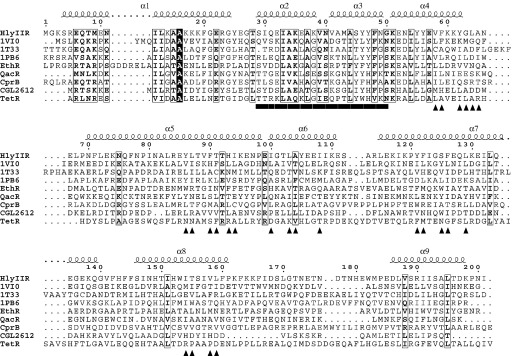
Multiple sequence alignment of HlyIIR with several TetR family members for which the three-dimensional structures are available. Conserved residues are marked by boxes. The helix-turn-helix motif is underlined by a bar and triangles highlight residues forming the ligand-binding pocket. HlyIIR secondary structure elements are indicated above the sequence. Putative transcriptional repressors are named by their PDB code. The sequences were aligned using CLUSTAL W^35^ and the Figure was prepared using ESPript.^36^

The N-terminal domain of HlyIIR contains a three-helical bundle with helices α2 and α3 forming a typical helix-turn-helix DNA-binding motif found in 95% of prokaryotic transcriptional factors.^13^ Helix α1 stabilises the location and orientation of the HTH motif and also serves as an interface with the C-terminal domain. Residues of the N-terminal domain are conserved among the TetR family of repressors (Figure 2), except for helix α3 which is responsible for specific DNA sequence recognition. The three helices interact through a hydrophobic core, which is composed of highly conserved residues. The N-terminal half of helix α4 could be also considered part of the DNA-binding domain, as it contributes to the hydrophobic core and its primary sequence is conserved. The rest of helix α4 belongs to the C-terminal domain, which, in contrast to the N-terminal domain, is not conserved among the TetR family.

### C-terminal regulatory and dimerisation domain

The C-terminal domain of HlyIIR consists of six α-helices, five of which form an antiparallel bundle. The C-terminal domains of the two subunits of the dimer form a molecular core with 1790 Å^2^ of the surface area of each monomer buried in the contact area (17% of the total molecular surface).^14^ (Figures 1(c) and 4(b), below) Most intersubunit contacts are made by helices α6 and α8 with several additional interactions formed between the loop connecting helices α7 and α8 of one subunit and helix α9 of the second subunit of the dimer. Subunit–subunit interactions are largely hydrophobic. In addition, there are ten direct intersubunit hydrogen bonds and four hydrogen bonds mediated by water molecules. A region between helices α8 and α9 forms an “arm”, which enters a corresponding cavity on the surface of the second subunit of the dimer. The arm contains two consecutive α-helical turns. Part of the arm was not modelled because of the absence of clear electron density.

### Ligand binding pocket

**Figure 3:**
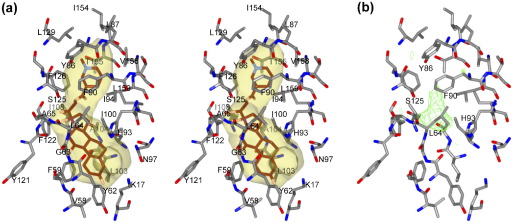
Ligand-binding pocket. (a) Stereo view with the cavity contoured as in Figure 1(c) and cavity-lining residues shown by sticks. The compound that gave a highest score during the AutoDock screening (NCI 23904) is shown with its carbon atoms in brown, oxygen atoms in pink and nitrogen atoms in light blue. (b) Difference electron density maps (green) corresponding to the pocket area and contoured at 3σ.

An important feature of the HlyIIR structure is a large internal cavity in the C-terminal α-helical bundle. This cavity is 18 Å long and has an internal volume of ∼ 550 Å^3^ (Figure 3). It is only partially filled by electron density (Figure 3(b)), which appears to correspond to solvent molecules or crystallisation buffer components, since mass-spectrometry analysis did not reveal any compounds bound to the protein (data not shown). Most of the inner surface of the cavity is lined by 25 hydrophobic residues (Figure 3(a)) contributed by helices α4–α8. It is likely that the entrance to the ligand binding pocket is formed by helices α4 and α5 and closed by side-chains of Tyr62 and His93 and main-chain atoms of helix α4.

The presence of this large cavity and analogy with the structural homologues suggest that DNA-binding properties of HlyIIR are modulated by interactions with a small-molecule ligand. Structural information for apo and ligand-bound forms is available for two members of the TetR family of repressors: QacR^18^ and TetR.^20^ In both cases, in the ligand-bound form, the DNA-binding domains are oriented differently to their position in the apo structure, explaining the lower affinity of the ligand-bound form towards the operator DNA. It is likely that the orientation of the DNA-binding domains of HlyIIR is also controlled by binding of a ligand.

In the structure of HlyIIR, helix α4 is followed by a residue segment Gly63–Phe71, which appears to be flexible as electron density is present only for its main-chain atoms. Secondary structure prediction^15^ and the presence of an equivalent long α-helix in the structures of other transcriptional repressors of the TetR family^13^ indicate that this segment is likely to form an extension of helix α4. It is thus possible that this segment is flexible in the absence of ligand and transforms into an α-helical conformation upon ligand binding. This structural rearrangement could be accompanied by movement of the DNA-binding domain. We probed the potential ability of the DNA-binding domain to change its position by Normal Mode Analysis calculations performed by ElNemo.^37^ This analysis showed low energy modes corresponding to considerable rotation of the DNA-binding domain with positional shifts of up to 7 Å in helix α3 observed with default ElNemo parameters (Supplementary Data, movie 1).

### Virtual ligand library screening

Since a remarkably large hydrophobic cavity was observed in the structure of HlyIIR, virtual ligand screening was performed to understand the nature of small molecules that could bind to HlyIIR. The NCI Diversity Set ligand library, screened by AutoDock^16^ using the Lamarckian genetic algorithm, resulted in predicted binding free energies for different compounds ranging from − 16.7 to + 25 kcal/mol. As a positive control, dockings of native QacR substrates (Etidium, Malachite Green, Rhodamine Q) to the QacR structure resulted in predicted binding free energies ranging from − 12.9 to − 9.7 kcal/mol. The top five compounds selected from the NCI Diversity Set, which had the lowest predicted binding free energies (from − 16.7 to − 14.6 kcal/mol), were steroid derivatives containing additional methyl and hydroxyl groups connected to the steroid core and an additional aromatic ring connected to the core by a long linker chain. The selected compounds differ in composition of the linker chain and the chemistry of the aromatic ring. The compound with the lowest predicted binding free energy (NCI 23904) is shown on Figure 3(a). It fills most of the HlyIIR ligand-binding pocket without any clashes with the protein atoms. We conclude that the HlyIIR pocket is able to accommodate a relatively large ligand, with a molecular mass of up to 400–500 Da.

## Discussion

*B. cereus* can produce several extracellular enzymatic and pore-forming protein toxins. Synthesis of many of them is induced by the global regulator PlcR^5^ when bacilli enter a stationary phase. In contrast, expression of the gene for pore-forming cytotoxin hemolysin II starts in the middle of logarithmic phase and is under more complex genetic control involving at least two protein factors: the global regulator Fur and the specific regulator HlyIIR.

A search for HlyIIR structural homologues by DALI^17^ resulted in a number of hits to members of the TetR family of repressors which are key players in virulence, multi-drug resistance, pathogenicity processes and are responsible for rapid adaptation of the bacteria to changing environmental conditions.^13^ The top five structural homologues of HlyIIR, shown in Table 2, are non-characterized putative transcriptional regulators from *Bacillus subtilis, Salmonella typhimurium, E. coli, B. cereus* and *Rhodococcus sp.* whose structures were determined by structural genomics consortiums. In addition, the DALI search revealed strong structural similarity with several biochemically characterised members of the TetR family: QacR,^18^ CGL2612^19^ and TetR^20^ are transcriptional repressors controlling expression of drug efflux pumps, which render cells resistant to different antimicrobial compounds. EthR^21^ is a repressor for the gene encoding monooxygenase and CprB^22^ belongs to the γ-butyrolactone-type autoregulator/receptor system involved in cell–cell signalling.

**Figure 4:**
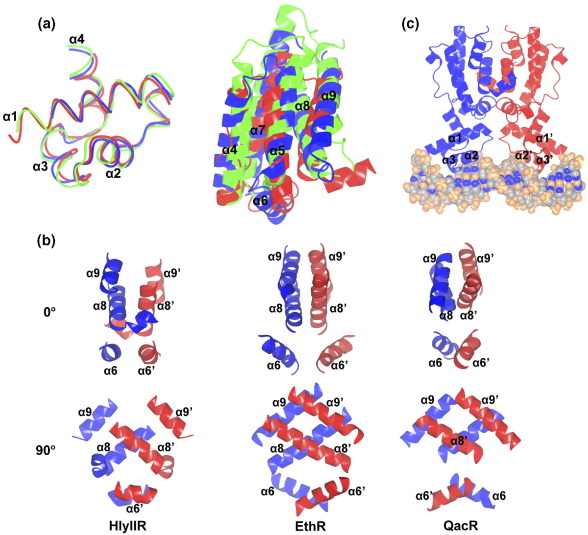
Structural comparison of HlyIIR with other members of the TetR family. (a) Superimposed N-terminal (left) and C-terminal (right) domains of HlyIIR (red) with QacR (PDB code 1JT0, blue) and EthR (1T56, green). (b) Architecture of the dimerisation interface in HlyIIR (left), EthR (middle) and QacR (right). Top and bottom: two views related by a 90° rotation around the vertical axis. Helices from two subunits of the dimer are shown in blue and red. (c) Schematic representation of the HlyIIR complex with the 22 bp *B*-form DNA. The DNA molecule is shown as a van der Waals model and the HlyIIR dimer is shown as ribbons.

Despite a very low overall sequence similarity of HlyIIR with its structural homologues (11% to 21% identity) the overall fold, including the number of helices, their length and relative spatial arrangement, is the same in all members of the TetR family. Assignment to this protein family is usually based on the N-terminal DNA-binding domain sequence conservation (PROSITE signature PS01081, Pfam profile PF00440; Figure 2).^13^ The structural and sequence variations in this domain appear to be constrained by its DNA-binding function. In accordance, the N-terminal 50 amino acid segment of HlyIIR displays ∼ 35% sequence identity and has a C^α^ atom r.m.s. deviation of 0.8 Å–1.5 Å with the other members of the TetR family listed in Table 2 (Figure 4(a), left).

The C-terminal domain displays no primary sequence conservation due to its adaptation to binding different ligand molecules and as a result the r.m.s. deviation calculated over the C^α^ atoms of structurally similar areas is in the range of 2.9 Å–4.7 Å (Figure 4(a), right). Despite the fact that in all regulators of the TetR family the ligand-binding pocket is located deep inside the C-terminal α-helical bundle, the entrance to the binding pocket is formed by different helices. For example, while the entrance into the ligand-binding site in QacR is located between helices α7 and α8 and the loop connecting α8′ and α9′ from the adjacent subunit of the dimer, in the structure of CGL2612 the deep cavity is enclosed between helices α4 and α5. The entrance to the ligand-binding pocket of EthR also differs: it opens at the top of the molecule between helices α4, α5 and α7.^18,19,21^ Similarly to CGL2612, the entrance to the ligand-binding pocket of HlyIIR appears to be formed by helices α4 and α5.

The homodimerisation interface in members of the TetR family is usually formed by helices α6, α8 and α9. Depending on the particular protein, the spatial arrangement of these helices (Figure 4(b)) and structural details of subunit–subunit interactions differ, in spite of the conserved fold. For instance, in the structures of HlyIIR homologues helix α9 makes multiple strong contacts with helix α9′ of the second subunit of the dimer, while in the HlyIIR structure helix α9 is shifted and forms only a few inter-subunit contacts; these are made with the loop connecting helices α7′ and α8′ of the second subunit.

The structural similarity of HlyIIR with members of the TetR family suggests a similar mode of interaction with DNA. On the basis of a 3D structural alignment of HlyIIR with QacR-DNA and TetR–DNA complexes^23,24^ and in accordance with the Suzuki rules^25^ we propose a model for the complex of HlyIIR with *B*-form DNA, where specific recognition of nucleotide sequences is achieved by fitting helices α3 and α3′ from the two HTH motifs of the dimer into the major groove of the DNA (Figure 4(c)). The analysis suggests that amino acids Val41, Ala42, Ser45 and Tyr46 from helix α3 form specific contacts with nucleotides in DNA major groove, while Asn40 and Lys51, located at opposite ends of helix α3, are likely to interact with the DNA backbone phosphate groups. The distance between the two DNA-recognition helices of the HlyIIR dimer is about 35 Å, meaning that the two helices fitting into the major groove are separated by one full turn of the DNA. In such a complex the protein dimer covers about 20 bp of the DNA. As HlyIIR specifically protected ∼ 50 bp of the *hlyII* operator DNA from DNase I digestion,^8^ at least two dimers of HlyIIR should bind to one *hlyII* operator region. This hypothesis is in agreement with biochemical data obtained by fluorescence anisotropy (Rodikova *et al.*, unpublished results). HlyIIR homologues change the DNA structure during binding, for instance TetR bends DNA, while QacR transforms *B*-form DNA into an under-twisted configuration.^23,24^ However, CD spectra indicate no considerable structural changes in DNA upon binding to HlyIIR (Rodikova *et al*., unpublished results). The full understanding of structural events that occur during binding of DNA to HlyIIR must await structure determination of the HlyIIR/DNA complex.

Available genome sequences suggest that *hlyII* and *hlyIIR* genes are present in all bacteria of the *B. cereus* group except for *B. cereus* ATCC 10987. Interestingly, the hemolysin II gene is disrupted by a frame-shift mutation in all available *B. anthracis* genomes, though the *hlyIIR* gene has only two nucleotide substitutions resulting in single amino acid change of Gly2 to Glu2. This residue is located within a disordered part of the HlyIIR structure and is not significant for the DNA or ligand binding. In contrast, comparison of the HlyIIR from *B. cereus* strain B771, used for this work, with HlyIIR from the standard strain ATCC 14579 reveals 11 amino acid substitutions. All except for one of these substitutions appear to be insignificant, since the mutated amino acid residues are located on the protein surface and are unlikely to influence protein function. The only observed substitution that might affect HlyIIR function is Ser125 to Asn125. This residue contributes to the formation of the ligand-binding pocket, therefore its mutation could alter ligand-binding specificity.

Our data strongly suggest that the repressor activity of HlyIIR is modulated by a small-molecule ligand. It is difficult to predict precisely the composition of this ligand from the structure of the protein cavity alone, since the geometry and dimensions of the ligand-binding pocket may undergo significant changes during ligand binding, as previously shown for QacR.^18^ Nevertheless, the virtual screening approach allowed an estimation of the approximate size and prediction of the overall geometry of the HlyIIR ligand (Figure 3(a)). The largely hydrophobic character of residues lining the ligand-binding pocket with very few hydrogen bond donors/acceptors and no charged residues, suggests that the natural ligand has a hydrophobic character. The top five compounds selected from the NCI Diversity Set are all steroid derivatives, which are unlikely related to natural processes in the bacterial cell. HlyIIR is a regulator for the pore-forming toxin, which forms a transmembrane channel and therefore damages mammalian cells. It is interesting that the top selected compounds from virtual screening are structurally very close to cholesterol, a major component of animal cell membranes. Docking of a cholesterol molecule gives a predicted binding free energy of − 12.8 kcal/mol, comparing favourably with docking experiments performed for QacR and its natural ligands. It is thus possible that a real HlyIIR ligand is a cholesterol derivative or a product of cholesterol processing/degradation by bacterial enzymes.

Preliminary data obtained by the genomic systematic evolution of ligands by exponential enrichment (SELEX) method show that *in vitro* HlyIIR binds promoter regions of at least four genes in addition to the *hlyII* gene (Rodikova *et al*., unpublished results), hence it appears to be a pleiotropic regulator involved in the regulation of different cellular processes. This observation is in agreement with the conservation of the *hlyIIR* gene in *B. anthracis*, where the hemolysin II gene is disrupted and thus HlyIIR may play other regulatory roles. Functional analysis of genes controlled by HlyIIR will help to identify its natural ligand(s). These and further biochemical studies are needed to understand the molecular logic of regulation by HlyIIR.

## Materials and Methods

### Expression and purification of HlyIIR

The previously reported plasmid pHR^8^ carrying the HlyIIR gene fused with the N-terminal 6-histidine tag was transformed into *E. coli* strain M15 (Qiagene). Cells were grown at 37 °C in LB medium supplemented with 100 μg/ml ampicillin. After reaching *A*_600_ = 0.6, cells were induced by addition of 1 mM isopropyl-β-d-thiogalactopyranoside (IPTG) followed by incubation for an additional 4 h. All subsequent steps were carried out at 4 °C. Cells were harvested by centrifugation, resuspended in buffer A (20 mM sodium-phosphate buffer (pH 7.5), 1 M NaCl, 20 mM imidazole) with protease inhibitor AEBSF (to a final concentration of 1 mM) and lysozyme (to a final concentration of 0.1 mg/ml), followed by sonication. The lysate was clarified by ultracentrifugation and the supernatant was loaded into a HisTrap HP column (Amersham Biosciences) charged with Ni^2+^ and equilibrated with buffer A. Protein was eluted using a linear gradient (20 mM–300 mM) of imidazole in the same buffer. HlyIIR-containing fractions were pooled, concentrated using a Centricon 10K concentrator (Millipore) and subjected to gel-filtration chromatography using a Superdex 75 column (Amersham Biosciences) equilibrated in buffer containing 20 mM sodium-phosphate (pH 7.5), 1 M NaCl, 5 mM EDTA. Fractions containing homogenous HlyIIR (as judged by SDS–PAGE) were pooled, concentrated to 20 mg/ml using a Centricon 10K concentrator, aliquoted, flash-frozen in liquid nitrogen and stored at − 80 °C.

### Expression of HlyIIR selenomethionine derivative

For preparation of selenomethionine (SeMet) HlyIIR, the methionine auxotroph *E. coli* strain B834 (DE3)-pLysS (Novagen) was transformed with plasmid pHR.^8^ We used 1 ml of an overnight culture to inoculate 50 ml of LB medium supplemented with 100 μg/ml of ampicillin and grown at 37 °C to *A*_600_ = 1.0. The cells were harvested by centrifugation, washed three times and used to inoculate 1 l of the SeMet growth medium, which comprises 2 × M9 medium, 0.4% (w/v) glucose, 2 mM MgSO_4_, 25 μg/ml FeSO_4_.7H_2_O and 40 mg/l of all the amino acids except for Met, which was replaced by SeMet. The growth medium was supplemented with vitamins (riboflavin, niacinamide, pyridoxine monohydrochloride and thiamine, each at 1 mg/ml) and 100 μg/ml of ampicillin. Expression of SeMet-HlyIIR was induced at an *A*_600_ of 0.7 by the addition of 1 mM IPTG. After 6 h of further growth the cells were harvested and SeMet-substituted HlyIIR was purified by the same protocol described for the native protein. MALDI-TOF mass spectrometry of native and SeMet-derivatized HlyIIR indicated complete incorporation of SeMet.

### Crystallisation and data collection

Crystals of native HlyIIR protein were obtained by the hanging drop vapour diffusion method by mixing equal amounts of protein sample with a reservoir solution containing 100 mM sodium cacodylate buffer (pH 6.0), ammonium sulphate at 60% saturation and 3%(v/v) isopropanol. Crystals grew over five to ten days at 20 °C to a size of 0.1 mm × 0.1 mm × 0.4 mm. Under similar conditions, the selenomethionine derivative of HlyIIR formed an amorphous precipitate. SeMet-HlyIIR crystals were obtained with ammonium sulphate (50% saturation) and 100 mM sodium acetate (pH 5.0) in the reservoir and with addition of 3 mM TCEP in the protein solution. Crystals grew for 20 days at 20 °C to a size of 0.1 mm × 0.1 mm × 1 mm. Prior to data collection, crystals were transferred to a cryoprotecting solution containing all reservoir solution components supplemented with 20%(v/v) glycerol and flash frozen in liquid nitrogen. Crystals of native HlyIIR and its SeMet derivative had similar cell dimensions and contained one monomer of HlyIIR per asymmetric unit resulting in a solvent content of ∼ 67%.

X-ray data at three different wavelengths from a single crystal of SeMet-HlyIIR were collected at the BM14 beamline, ESRF, Grenoble. An X-ray fluorescence spectrum was recorded and used to choose the optimal wavelengths for MAD data collection at three wavelengths: 0.9794 Å (*f*′ minimum), 0.9792 Å (*f*″ maximum) and 0.9184 Å (remote high-energy wavelength). Each data set was processed using DENZO and SCALEPACK.^26^

### Structure solution and refinement

Initial phases were obtained by SOLVE^27^ followed by density modification and automated model building by RESOLVE.^28^ This led to an initial model containing 74% of amino acid residues, 46% of which contained side chains (*R*_work_ = 37.4%, *R*_free_ = 41.0%). The model was gradually completed through several cycles of model building by Coot^29^ and QUANTA (Quanta2005, Accelrys Software Inc.) followed by refinement with REFMAC5.^30^ Solvent atoms were initially built using the program ARP/wARP^31,32^ and later added or removed by manual inspection. The final refinement cycles utilised translation-libration-screw-motion (TLS) parameters and resulted in *R*_work_ and *R*_free_ values of 22.3% and 27.6%, respectively, calculated for 13,895 reflections observed in a 2.4 Å–25 Å resolution range (Table 1). The side-chains of four lysine residues were poorly defined in the electron density maps and their occupancy was set to 0.01. Residues 170–185 were not modelled because of the lack of clear electron density. The model was analysed for stereochemical quality using PROCHECK.^12^ Statistics of structure determination and refinement are shown in Table 1.

### Virtual ligand library screening using AutoDock

The AutoDock 3.0 software package^16^ was used for virtual screening of NCI Diversity Set compounds. The docking area was assigned visually to cover the internal HlyIIR cavity. A grid of 40 Å × 40 Å × 40 Å with 0.375 Å spacing was calculated around the docking area for all atom types presented in the NCI Diversity Set using AutoGrid. An AutoDock-ready version of the NCI Diversity Set available on the official AutoDock web site was used. For each ligand 20 separate docking calculations were performed. Each docking calculation was limited to 750,000 energy evaluations using the Lamarckian genetic algorithm local search and was performed with a population size of 150, a mutation rate of 0.02 and a crossover rate of 0.8. The results were ranked on the basis of predicted free energy of binding.

### Protein Data Bank accession code

Coordinates and structure factors for the structure of HlyIIR protein have been deposited at the RCSB Protein Data Bank (PDB) with accession code 2FX0.

## Figures and Tables

**Supplementary figure:**
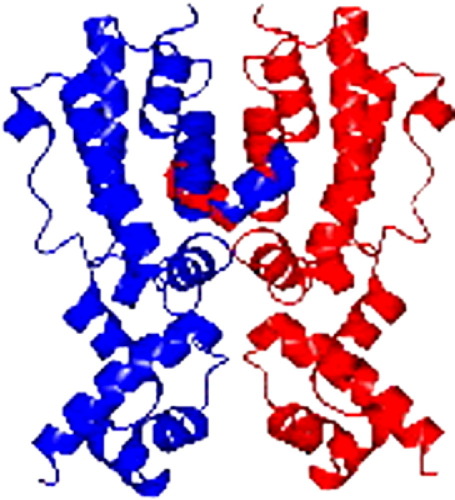


**Table 1:** Crystallographic statistics

A. *Data collection*			
Space group	*P*6_1_22		
Unit cell dimensions			
*a*, *b* (Å)	124.1		
*c* (Å)	79.5		
Wavelength (Å)	0.9792 (peak)	0.9794 (inflection)	0.9184 (remote)
*f*′, *f*″ (e)	− 8.09, 6.39	− 9.87, 3.41	− 4.1, 3.5
Resolution (Å) (outer shell)	25–2.4 (2.49–2.4)	25–2.7 (2.8–2.7)	25–2.9 (3.0–2.9)
Unique reflections	14,593 (1422)	10,250 (874)	8440 (800)
Redundancy^a^	11.5 (10.5)	6.6 (5.1)	6.9 (7.2)
Completeness (%)	99.9 (99.3)	98.5 (87.1)	99.8 (99.8)
<*I*/σ(*I*)>	21.5 (3.8)	21.7 (3.4)	18.2 (3.9)
*R*_merge_^b^ (%)	9.0 (58.8)	7.3 (42.9)	10.2 (55.7)

B. *Structure refinement*			
Resolution range (Å)	25–2.4		
No. of reflections in refinement	13,895		
*R*-factor^c^ (%)	22.3		
No. of reflections used for *R*_free_	695		
Free *R-*factor^c^ (%)	27.6		
No. of protein atoms	1476		
No. of water molecules	114		
Wilson *B*-factor (Å^2^)	45.3		
Average *B*-factor (Å^2^)	51.9		

*r.m.s. deviations*			
Bond lengths^d^ (Å)	0.012 (0.02)		
Bond angles^d^ (deg.)	1.17 (2.0)		

*Ramachandran statistics*			
Most favoured regions (%)	91.3		
Additionally allowed regions (%)	8.7		
Generously allowed and disallowed regions (%)	0		

a The average number of observations of the same reflection.

b The value of the merging *R* factor between equivalent measurements of the same reflection, *R*_I_ = ∑|*I*–<*I*>|/∑ *I*.

c Crystallographic *R*-factor, *R*_(free)_ = ∑||*F*_o_|–|*F*_c_||/∑|*F*_o_|.

d r.m.s. deviation from the standard values is given with target values in parentheses.

**Table 2:** Structural homologs of HlyIIR according to DALI^17^

Protein	Details	PDB code	*Z-*score	C^α^ r.m.s.d.	Identity (%)	Aligned residues	Protein length
Yer0, *Bacillus subtilis*	Non-characterized, structural genomics	1vi0	14	3.1	22	164	184
Putative TetR family repressor, *Salmonella typhimurium*	Non-characterized, structural genomics	1t33	13.6	3	19	167	220
Putative TetR family repressor, *Bacillus cereus*	Non-characterized, structural genomics	2fq4	13.2	4.7	21	164	183
YcdC, *Escherichia coli*	Non-characterized, structural genomics	1pb6	13.1	3.5	22	170	198
Putative TetR family repressor, *Rhodococcus sp*.	Non-characterized, structural genomics	2g3b	12.6	4.1	16	158	185
QacR, *Staphylococcus aureus*	Multi-drug resistance	1jty	12.5	4.6	21	165	186
CprB (ArpA-like), *Streptomyces coelicolor*	Cell-cell signalling	1ui5	12.3	2.9	21	160	195
EthR, *Mycobacterium tuberculosis*	Ethionamide resistance	1t56	12.3	3.1	11	160	193
CGL2612, *Corynebacterium glutamicum*	Drug resistance-related	1v7b	9.8	4	12	152	175
TetR, *Escherichia coli*	Tetracycline resistance	2tct	8.4	4	12	147	198

## References

[1] Ash C., Farrow J.A., Dorsch M., Stackebrandt E., Collins M.D. (1991). Comparative analysis of *Bacillus anthracis*, *Bacillus cereus*, and related species on the basis of reverse transcriptase sequencing of 16 S rRNA. Int. J. Syst. Bacteriol..

[2] Kramer J.M., Gilbert R.J., Doyle M.P. (1989). *Bacillus cereus* and other *Bacillus* species. Foodborne Bacterial Pathogens.

[3] Garcia Arribas M.L., Plaza C.J., de la Rosa M.C., Mosso M.A. (1988). Characterization of *Bacillus cereus* strains isolated from drugs and evaluation of their toxins. J. Appl. Bacteriol..

[4] Granum P.E., Doyle M., Beuchat L., Montville T. (2001). *Bacillus cereus*. Food Microbiology. Fundamentals and Frontiers.

[5] Slamti L., Lereclus D. (2002). A cell-cell signaling peptide activates the PlcR virulence regulon in bacteria of the *Bacillus cereus* group. EMBO J..

[6] Ivanova N., Sorokin A., Anderson I., Galleron N., Candelon B., Kapatral V. (2003). sequence of *Bacillus cereus* and comparative analysis with *Bacillus anthracis*. Nature.

[7] Harvie D.R., Vilchez S., Steggles J.R., Ellar D.J. (2005). *Bacillus cereus* Fur regulates iron metabolism and is required for full virulence. Microbiology.

[8] Budarina Z.I., Nikitin D.V., Zenkin N., Zakharova M., Semenova E., Shlyapnikov M.G. (2004). A new *Bacillus cereus* DNA-binding protein, HlyIIR, negatively regulates expression of *B. cereus* haemolysin II. Microbiology.

[9] Song L., Hobaugh M.R., Shustak C., Cheley S., Bayley H., Gouaux J.E. (1996). Structure of staphylococcal alpha-hemolysin, a heptameric transmembrane pore. Science.

[10] Andreeva Z.I., Nesterenko V.F., Yurkov I.S., Budarina Z.I., Sineva E.V., Solonin A.S. (2006). Purification and cytotoxic properties of *Bacillus cereus* hemolysin II. Protein Expr. Purif..

[11] Altschul S.F., Madden T.L., Schaffer A.A., Zhang J., Zhang Z., Miller W., Lipman D.J. (1997). Gapped BLAST and PSI-BLAST: a new generation of protein database search programs. Nucl. Acids Res..

[12] Laskowski R.A., MacArthur M.W., Moss D.S., Thornton J.M. (1993). PROCHECK: a program to check the stereochemical quality of protein structures. J. Appl. Crystallog..

[13] Ramos J.L., Martinez-Bueno M., Molina-Henares A.J., Teran W., Watanabe K., Zhang X. (2005). The TetR family of transcriptional repressors. Microbiol. Mol. Biol. Rev..

[14] Jones S., Thornton J.M. (1996). Principles of protein-protein interactions. Proc. Natl Acad. Sci. USA.

[15] Cuff J.A., Barton G.J. (1999). Evaluation and improvement of multiple sequence methods for protein secondary structure prediction. Proteins: Struct. Funct. Genet..

[16] Morris G.M., Goodsell D.S., Halliday R.S., Huey R., Hart W.E., Belew R.K., Olson A.J. (1998). Automated docking using a Lamarckian genetic algorithm and and empirical binding free energy function. J. Computat. Chem..

[17] Holm L., Sander C. (1998). protein fold space with Dali/FSSP. Nucl. Acids Res..

[18] Schumacher M.A., Miller M.C., Grkovic S., Brown M.H., Skurray R.A., Brennan R.G. (2001). Structural mechanisms of QacR induction and multidrug recognition. Science.

[19] Itou H., Okada U., Suzuki H., Yao M., Wachi M., Watanabe N., Tanaka I. (2005). The CGL2612 protein from *Corynebacterium glutamicum* is a drug resistance-related transcriptional repressor: structural and functional analysis of a newly identified transcription factor from genomic DNA analysis. J. Biol. Chem..

[20] Kisker C., Hinrichs W., Tovar K., Hillen W., Saenger W. (1995). complex formed between Tet repressor and tetracycline-Mg^2+^ reveals mechanism of antibiotic resistance. J. Mol. Biol..

[21] Dover L.G., Corsino P.E., Daniels I.R., Cocklin S.L., Tatituri V., Besra G.S., Futterer K. (2004). Crystal structure of the TetR/CamR family repressor *Mycobacterium tuberculosis* EthR implicated in ethionamide resistance. J. Mol. Biol..

[22] Natsume R., Ohnishi Y., Senda T., Horinouchi S. (2004). Crystal structure of a gamma-butyrolactone autoregulator receptor protein in *Streptomyces coelicolor* A3(2). J. Mol. Biol..

[23] Schumacher M.A., Miller M.C., Grkovic S., Brown M.H., Skurray R.A., Brennan R.G. (2002). Structural basis for cooperative DNA binding by two dimers of the multidrug-binding protein QacR. EMBO J..

[24] Orth P., Schnappinger D., Hillen W., Saenger W., Hinrichs W. (2000). Structural basis of gene regulation by the tetracycline inducible Tet repressor-operator system. Nature Struct. Biol..

[25] Suzuki M., Yagi N. (1994). DNA recognition code of transcription factors in the helix-turn-helix, probe helix, hormone receptor, and zinc finger families. Proc. Natl Acad. Sci. USA.

[26] Otwinowski Z., Minor W. (1997). Processing of X-ray diffraction data collected in oscillation mode. Methods in Enzymology. part A.

[27] Terwilliger T.C., Berendzen J. (1999). Automated MAD and MIR structure solution. Acta Crystallog. sect. D.

[28] Terwilliger T.C. (2000). Maximum-likelihood density modification. Acta Crystallog. sect. D.

[29] Emsley P., Cowtan K. (2004). Coot: model-building tools for molecular graphics. Acta Crystallog. sect. D.

[30] Murshudov G.N., Vagin A.A., Dodson E.J. (1997). Refinement of macromolecular structures by the maximum-likelihood method. Acta Crystallog. sect. D.

[31] Perrakis A., Morris R., Lamzin V.S. (1999). Automated protein model building combined with iterative structure refinement. Nature Struct. Biol..

[32] Collaborative Computational Project, Number 4 (1994). The CCP4 suite: programs for protein crystallography. Acta Crystallog. sect. D.

[33] Laskowski R.A. (1995). SURFNET: a program for visualizing molecular surfaces, cavities, and intermolecular interactions. J. Mol. Graph..

[34] Potterton E., McNicholas S., Krissinel E., Cowtan K., Noble M. (2002). The CCP4 molecular-graphics project. Acta Crystallog. sect. D.

[35] Thompson J.D., Higgins D.G., Gibson T.J. (1994). CLUSTAL W: improving the sensitivity of progressive multiple sequence alignment through sequence weighting, position-specific gap penalties and weight matrix choice. Nucl. Acids Res..

[36] Gouet P., Courcelle E., Stuart D.I., Metoz F. (1999). ESPript: analysis of multiple sequence alignments in PostScript. Bioinformatics.

[37] Suhre K., Sanejouand Y.H. (2004). ElNemo: a normal mode web-server for protein movement analysis and the generation of templates for molecular replacement. Nucl. Acids Res..

